# Surface colonization of *Aureobasidium pullulans*: a multimodal microscopy study toward living coating development

**DOI:** 10.1038/s41598-026-48101-5

**Published:** 2026-04-08

**Authors:** Ihab Malat, Anja Černoša, Anna Sandak

**Affiliations:** 1 InnoRenew CoE, Andrej Marušič Institute, University of Primorska, Muzejski trg 2, Koper, 6000 Slovenia; 2 InnoRenew CoE, University of Primorska, Faculty of Mathematics, Natural Sciences and Information Technologies, Glagoljaška ulica 8, Koper, 6000 Slovenia

**Keywords:** *Aureobasidium pullulans*, Surface colonization, Plastic, Pine wood, Living coating, Biological techniques, Biotechnology, Materials science, Microbiology

## Abstract

Recent advances in engineered living materials (ELMs), which integrate living cells into functional structural and protective systems, have accelerated interest in understanding microbial surface interactions at fundamental scales. In this context, *Aureobasidium pullulans*, a polymorphic and polyextremotolerant black yeast fungus, has emerged as a promising candidate for diverse biotechnological applications, including engineered living coatings. However, its microscale surface colonization dynamics remain insufficiently characterized, limiting predictive control over colonization of various surfaces. Complementary optical approaches, including VHX digital imaging, fluorescence observation, and quantitative fluorescence measurements, were used to characterize temporal colonization of fungus on plastic coverslips and pine wood, representing two contrasting substrates used in the building sector. On plastic substrates, initial attachment involved dispersed cells that rapidly proliferated and merged into a continuous layer. Quantitative fluorescence revealed a significant increase in signal from Day 1 to Day 3 (from 25,328 to 42,510 RFU; *P* = 5.4 × 10⁻⁴) followed by a decrease by Day 6 (10,555 RFU). This decline may be associated with reduced dye penetration into the compact, melanized matrix rather than a reduction in biomass. On wood, colonization followed the native fiber orientation and progressed into cohesive multicellular structures. The results provide a new understanding of substrate-dependent colonization of *A. pullulans* and highlight methodological limitations in quantifying biomass on porous, heterogeneous materials. The multimodal microscopy framework established a robust comparative platform for analysing fungal-material interactions and enabled the rational development of fungal-based living coatings for protective and functional applications.

## Introduction

*Aureobasidium pullulans* is a polymorphic black yeast belonging to the family *Dothioraceae*^1^. It is widely recognized for its ability to synthesize pullulan, an extracellular polysaccharide with broad applications in the pharmaceutical, biomedical, and food industries^2,3^. Beyond its biotechnological value, *A. pullulans* is frequently detected on natural and artificial surfaces, including plant tissues, wood, and indoor materials^4^, suggesting a strong capacity for surface-associated growth. Depending on environmental conditions, the species exhibits multiple morphological forms ranging from yeast-like blastoconidia to filamentous hyphae and melanized arthroconidia^5^, which may contribute to its persistence at solid surfaces. Genomic analyses further have identified genes associated with stress tolerance, adhesion, and extracellular matrix production^4,6^, suggesting potential functional traits that may support adaptation to fluctuating surface environments.

In response to increasing demands for sustainable and low-impact material protection, living coatings have emerged as an innovative alternative to conventional chemical formulations^7,8^. Unlike traditional coatings, which rely on passive barriers that degrade over time, living coatings incorporate metabolically active microorganisms capable of self-repair, adaptive response, and long-term functional renewal^7,8^. These systems can persist under fluctuating environmental conditions, regenerate damaged areas, and continuously produce protective biopolymers, offering extended durability and reduced maintenance compared with synthetic coatings^7,8^. In practical applications, the long-term functionality of living coatings depends on the availability of nutrients to sustain microbial activity^9^. Under nutrient-limited conditions, cells may enter a dormant state, reducing metabolic activity and self-regeneration capacity^10^. Strategies to overcome this limitation include designing systems that utilize environmental nutrients (e.g., airborne organic compounds), incorporating slow-release substrates, or enabling periodic reactivation under favorable conditions^11^. Moreover, the selection of oligotrophic microorganisms, such as *A. pullulans* constitutes a promising strategy to mitigate nutrient limitations, given their metabolic versatility and broad enzymatic profile, which enable sustained functionality in low resource environments^6^. Recent studies have demonstrated the use of microbial coatings for wood protection, self-healing materials, and biofunctional surfaces, highlighting both their potential and the need to better understand their behavior under realistic environmental constraints^12^.

In this context, *A. pullulans* represents a particularly promising candidate for such applications due to its pronounced extremotolerance, strong surface-colonization ability, and production of extracellular polysaccharides that enhance adhesion and interface stability^4,13^. Its natural habitat suggests an ability to establish persistent surface-associated communities^4^. Importantly, *A. pullulans* are considered non-pathogenic and are widely regarded as safe for both environmental and biotechnological uses, with several strains already employed in food, agricultural, and industrial applications^5^. These characteristics position *A. pullulans* as a biologically compatible and ecologically robust organism suitable for next generation living coating technologies.

In microbial systems, biofilms consist of a surface-attached community of microorganisms embedded in a self-produced extracellular polymeric substance (EPS) matrix that provides structural stability and mediates adhesion to interfaces^9,14^. Biofilm development generally follows a conserved sequence: initial reversible attachment, irreversible adhesion supported by EPS secretion, microcolony formation, three-dimensional maturation, and eventual dispersal^9,14^. Most current knowledge of biofilm development comes from bacterial model systems such as *Pseudomonas aeruginosa*, *Staphylococcus aureus*, and *Bacillus subtilis*^15–18^. These systems have shaped the methodological toolbox, including microtiter plate biomass assays (e.g., crystal violet assay)^19^, confocal laser scanning microscopy^20^, polymerase chain reaction targeting known biofilm-related genes (e.g., those for quorum sensing, adhesins, or exopolysaccharide production)^21^ and surface-interaction analyses at biointerfaces^22^. Among fungi, *Candida albicans*, *Aspergillus fumigatus*, and *Cryptococcus neoformans* are the most intensively studied biofilm formers, known for forming dense hyphal networks or yeast-hyphae mixed structures with robust EPS layers that limit antimicrobial penetration^23–25^. Most analytical approaches used for fungal biofilms are adapted from bacterial biofilm research^26^. However, significant physiological and structural differences between bacteria and fungi mean that some techniques require modification, and certain approaches are specific to each organism. For example, widely used bacterial assays such as crystal violet staining or confocal laser scanning microscopy with shallow optical penetration often underestimate fungal biomass or fail to resolve the three-dimensional hyphal networks that characterize filamentous fungi^27^. As a result, fungal biofilms remain under characterized, particularly during early adhesion and structural organization.

Despite extensive ecological and physiological characterization, little is known about how *A. pullulans* initiates biofilm formation and adheres to solid substrates. Existing studies have focused primarily on the macroscopic development of biofilm on wood during long-term outdoor exposure, demonstrating their persistence and protective function^12,28,29^, but without resolving microscale colonization patterns or temporal progression. To date, no time-resolved or quantitative methodology has been applied to characterize *A. pullulans* adhesion across different substrates, leaving key aspects of adhesion behavior and substrate dependence unexplored. Understanding how *A. pullulans* interact with different interfaces is therefore essential for both fundamental biointerface science and the development of biologically based surface technologies.

This study addresses this gap by characterizing the early surface colonization of *A. pullulans* on two contrasting substrates: plastic, a smooth, non-porous polyester-based material representative of synthetic surfaces commonly found in built environments^30^, and pine wood, a porous, anisotropic, and hydrophilic lignocellulosic material widely used in sustainable construction and various outdoor structures^31^. These materials differ substantially in chemistry, roughness, and moisture retention, providing complementary models for understanding fungal interactions with natural and engineered interfaces.

Fungal associated biomass on plastic coverslips was assessed using fluorescence imaging and quantification with Calcofluor White staining, while colonization on pine wood was examined using fluorescence imaging to resolve spatial organization across the heterogeneous lignocellulosic surface. Assessing substrate-dependent fungal colonization responses provides insight into the temporal dynamics and interfacial behavior of *A. pullulans*, supporting the design of stable and durable biological coating systems.

## Materials and methods

### Microorganism, media, and cell preparation

*Aureobasidium pullulans* strain EXF-3844 was obtained from the Culture Collection Ex of the Infrastructural Centre Mycosmo (Department of Biology, Biotechnical Faculty, University of Ljubljana, Slovenia), originally isolated from dried olives. The strain was maintained on Malt Extract Agar commercial mix (MEA; agar 15 g/L, malt extract 30 g/L, mycological peptone 5 g/L in distilled water; Sigma-Aldrich, St. Louis, MO, USA). Plates were incubated in a growth chamber at 25 °C for 5 days. For liquid cultivation, colonies were transferred into two 50 mL Falcon^®^ tube, the first contain 10 mL of Potato Dextrose Broth commercial mix (PDB; Dextrose 20 g/L, Potatoes Infusion from, 4 g/L in distilled water; Sigma-Aldrich) and the second contain 10 mL of Synthetic Nutrient Broth (SNB) composed of Potassium Dihydrogen Phosphate (KH_2_PO_4_,1 g/L; Sigma-Aldrich), Potassium nitrate (KNO_3_,1 g/L; Sigma-Aldrich), Magnesium sulfate heptahydrate (MgSO_4_ × 7H_2_O, 0.5 g/L; Sigma-Aldrich), Potassium chloride (KCl, 0.5 g/L; Sigma-Aldrich), glucose (0.2 g/L; Sigma-Aldrich), saccharose (0.2 g/L; Sigma-Aldrich) in distilled water. Cultures were incubated for 18 h at 25 °C with shaking at 180 rpm. Subsequently, 2 mL of the culture was transferred to a 2 mL Eppendorf tube and centrifuged at 10,000 × *g* for 10 min. The supernatant was discarded, and the pellet was resuspended in 2 mL Yeast Nitrogen Base (YNB) liquid medium (pH 7) composed of YNB (1.7 g/L; Sigma-Aldrich), Ammonium Sulfate ((NH_4_)_2_SO_4_, 5 g/L; Sigma-Aldrich) and glucose (20 g/L; Sigma-Aldrich) in distilled water. Cell suspensions were diluted using YNB liquid medium to reach optical densities (OD_600_) of 1.0 and 0.1, which served as standardized inoculum preparations for microscopy observations and surface colonization assays, respectively.

### Surface colonization and inoculation assays

#### *A. pullulans* colonization on plastic coverslips

**Coverslip preparation.** Nunc™ Thermanox™ coverslips (13 mm diameter; Thermo Scientific, Rochester, NY, USA) were placed in 24-well microtiter plates (Sarstedt, Nümbrecht, Germany) with the treated surface facing upward. A poly-L-lysine solution (1:10 in distilled water; Sigma-Aldrich) was sterile-filtered (0.22 μm) and applied (1 mL per well) for 30 min at room temperature to enhance cells adhesion. Coverslips were washed three times with Dulbecco’s Phosphate-Buffered Saline (DPBS; Sigma-Aldrich, St. Louis) and air-dried for 18 h at 25 °C.

**Inoculation, incubation and staining.** Cell suspension of *A. pullulans* was adjusted to OD_600_ 0.1 in YNB liquid medium. To each coverslip in wells 500 µL of *A. pullulans* cell suspension was added and then cultured for 18 h in SNB liquid medium. Control wells contained YNB liquid medium only. For each time point (1, 3, and 6 days), a total of 24 wells were prepared: 12 inoculated and 12 controls. Plates were incubated at 25 °C for the designated periods. After incubation, wells were gently rinsed twice with distilled water. Coverslips were stained with 500 µL of 0.3 mg/mL Calcofluor White (CFW; Sigma-Aldrich) for 90 min in the dark at 25 °C, followed by two additional washes with distilled water.

#### *A. pullulans* colonization on pine wood

**Wood preparation, inoculation and staining.** Autoclaved Scots pine wood (*Pinus sylvestris*) pieces (14 × 14 × 2 mm) were inoculated with 100 µL of *A. pullulans* suspension cultured for 18 h in PDB and OD_600_ adjusted to 0.1 in YNB liquid medium. A sterile cotton-tipped swab was used to distribute the suspension across the wood surface. Control pieces received YNB liquid medium only. Samples were incubated at 25 °C and 80% relative humidity for 1, 3, or 6 days. Three replicates of wood pieces inoculated by *A. pullulans* were used at each time point. After incubation, wood pieces were rinsed with distilled water, dried on Kimtech™ wipes (Kimberly-Clark Professional, Irving, TX, USA), and stained with 200 µL of 0.3 mg/mL CFW for 30 min in the dark at 25 °C. Samples were washed again with distilled water and dried before imaging.

### Imaging and analysis

#### Optical microscopy

Cells from OD_600_ 1 suspensions were air-dried on microscope slides and examined using a Leica DM2700 M upright optical microscope (Leica Microsystems, Wetzlar, Germany) under bright-field illumination. Images were acquired using objectives from ×40 to ×60, allowing resolution of yeast-like cells, filaments, and blastoconidia.

Surface colonization of *A. pullulans* cells on wood was examined using a VHX-6000 digital optical microscope (Keyence Corporation, Osaka, Japan). Images were acquired between ×20 and ×300 magnification to capture surface texture, biomass distribution, and interfacial coverage.

CFW-stained cells and surface colonization were visualized using an EVOS M7000 microscope (Thermo Fisher Scientific, Waltham, MA, USA) with DAPI filter (Ex 350–400 nm, Em 425–475 nm). Images were collected at magnifications ranging from ×10 to ×60 for coverslips and wood samples.

#### Fluorescence quantification

Fluorescence intensity of CFW-stained colonies was measured using a BioTek Synergy H1 microplate reader (Agilent Technologies, Santa Clara, CA, USA) held at 25 °C. Excitation and emission wavelengths were set at 360 nm and 440 nm, respectively. Full-surface scanning was enabled to ensure accurate RFU measurements for each well.

## Results

### Optical microscopy observation of *A. pullulans*

**Fig. 1:**
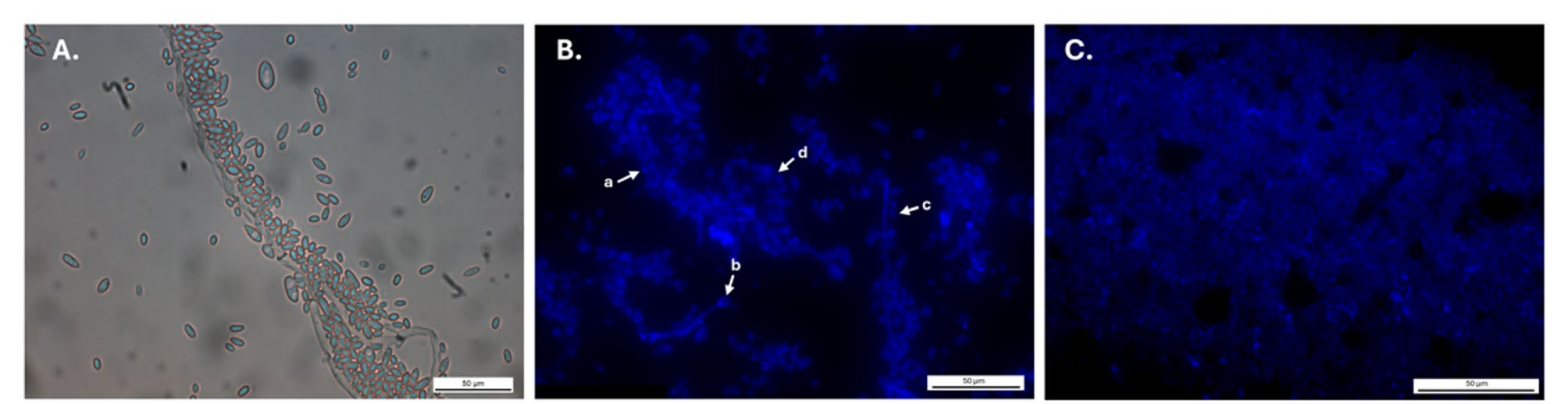
*A. pullulans* strain EXF-3844 observed by optical microscopy. (**A**) Optical (bright field) image acquired using a Leica DM2700 M microscope. (**B**,**C**) Fluorescence images acquired using an EVOS M7000 imaging system after CFW (0.3 mg/mL) staining. On panel (**B**) distinct fungal cell forms were observed (a) hyphae, (b) blastoconidia, (b) budding sites on hyphae, (d) yeast-like cell.

After 18 h of incubation, *A. pullulans* cells were examined using optical microscopy under bright-field and DAPI filter to characterize their early morphology before inoculation experiments. Under bright-field illumination (Fig. 1A), both elongated hyphal elements and numerous dispersed yeast-like cells were visible, with the hyphae appearing as continuous filamentous structures surrounded by oval cells of varying sizes. Fluorescence imaging with CFW (Fig. 1B) highlighted the cell walls of these structures, allowing clearer visualization of morphological variation. A hyphal segment was identified (a), along with several blastoconidia differing in size, including a small oval cell (b), a larger oval cell (c) and an elongated yeast-like cell (d). At a wider field of view, the fluorescence image (Fig. 1C) showed a dense and continuous layer of fungal structures covering the observed surface area.

### *A. pullulans* colonization on plastic coverslips

**Fig. 2:**
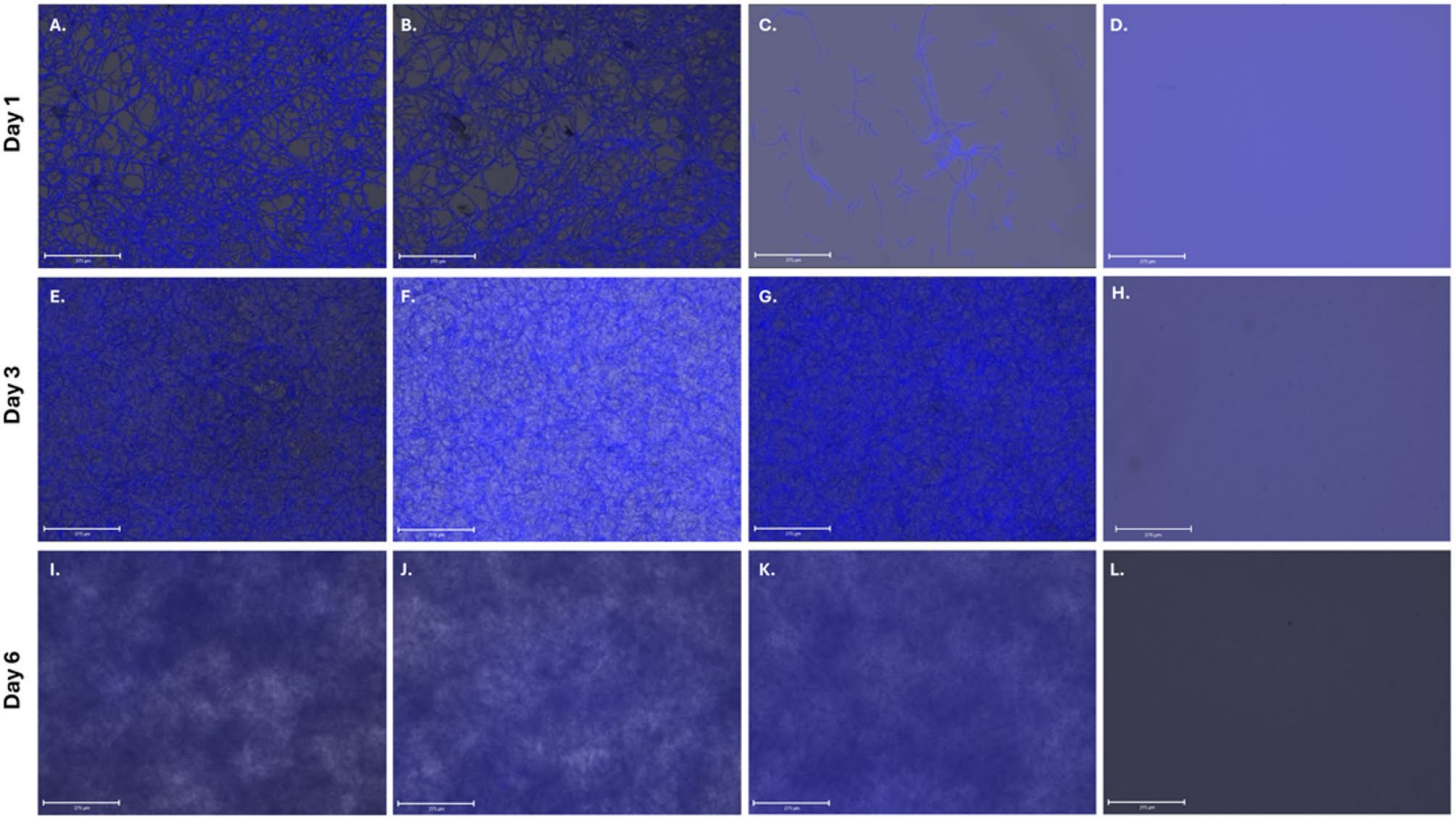
Fluorescent microscopy images of wells containing coverslips cultured with or without *A. pullulans* EXF-3844 over six days. Day 1 (**A**–**C**), day 3 (**E**–**G**), day 6 (**I**–**K**). Samples **D**, **H** and **L** represent controls for day 1, 3 and 6 respectively.

Calcofluor White fluorescence microscopy revealed a clear, time-dependent progression of surface colonization on plastic coverslips inoculated with *A. pullulans* (Fig. 2). At day 1, fluorescence images (Fig. 2; samples A-C) showed thin and discontinuous filamentous structures, with some fields displaying only sparse individual filaments or small clusters (Fig. 2; sample C), while others exhibited more interconnected networks (Fig. 2; samples A and B). By day 3, fluorescence intensity increased substantially, and a more homogeneous and continuous fluorescent layer was observed across the entire surface (Fig. 2; samples E-G), indicating denser colonization with limited variability between replicates. At day 6, the coverslips displayed diffuse and saturated fluorescence signals (Fig. 2; samples I-K), with minimal internal structural detail detectable, consistent with a compact and dense colonized layer. Across all time points, controls consistently appeared unchanged, whereas inoculated coverslips displayed progressively increased surface coverage in macroscopic and microscopic observations (Fig. 2; Samples D, H and L).

### *A. pullulans* surface biomass estimation

**Fig. 3:**
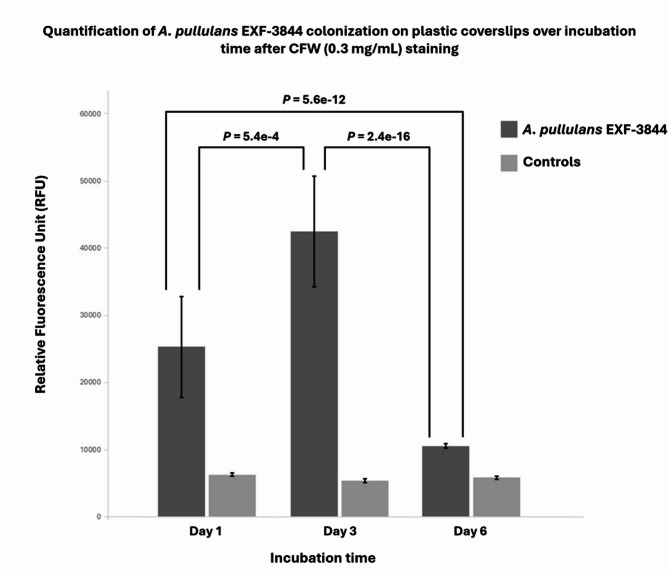
Quantification of *A. pullulans* EXF-3844 colonization on plastic coverslips over days 1, 3, and 6 after CFW (0.3 mg/mL) staining. Errors bars represent standard deviation between technical replicates of each day. *P* values represent the comparison between RFU means of different days.

Quantitative fluorescence measurements showed clear temporal differences in CFW signal intensity in treated wells and consistently low values in controls (Fig. 3). At day 1, treated wells displayed a mean of 25,328 ± 7,814 RFU, while controls remained low and uniform (6,293 ± 182 RFU). At day 3, treated wells increased to 42,510 ± 8,814 RFU, significantly higher than day 1 (*P* = 5.4 × 10⁻⁴). The SD ranges of day 1 (mean ± SD: 17,514–33,142 RFU) and day 3 (33,696–51,324 RFU) showed minimal boundary contact but no numerical overlap, indicating distinct intensity distributions between these time points. At day 6, treated fluorescence decreased to 10,555 ± 303 RFU and remained significantly lower than both day 1 (*P* = 5.6 × 10⁻¹²) and day 3 (*P* = 2.4 × 10⁻¹⁶). The day 6 range (10,252–10,858 RFU) did not overlap with either day 1 (17,514–33,142 RFU) or day 3 (33,696–51,324 RFU), confirming complete separation between these groups. In contrast, control wells showed tightly clustered values across days (day 1: 6,293 ± 182 RFU; day 3: 5,397 ± 152 RFU; day 6: 5,847 ± 210 RFU), with substantial overlap between their SD-based ranges (day 1: 6,111–6,475 RFU; day 3: 5,245–5,549 RFU; day 6: 5,637–6,057 RFU), and no significant differences were detected between control groups (*P* > 0.15 for all comparisons).

### *A. pullulans* colonization on pine wood

**Fig. 4:**
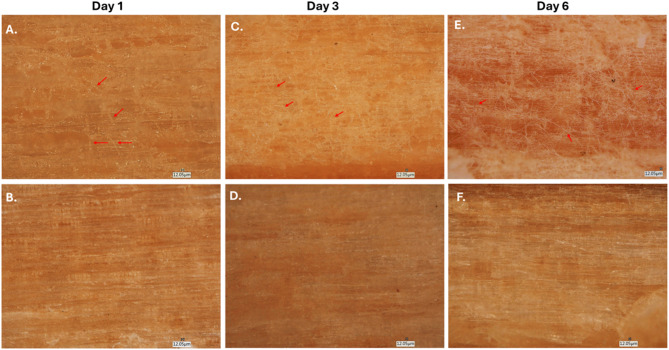
VHX macroscopic images of wood pieces inoculated with or without *A. pullulans* EXF-3844 over days 1, 3, and 6. for day 1 (with *A. pullulans* **A**; control **B**), day 3 (with *A. pullulans* **C**; control: **D**), day 6 (with *A. pullulans*: **E**; control: **F**).

**Fig. 5:**
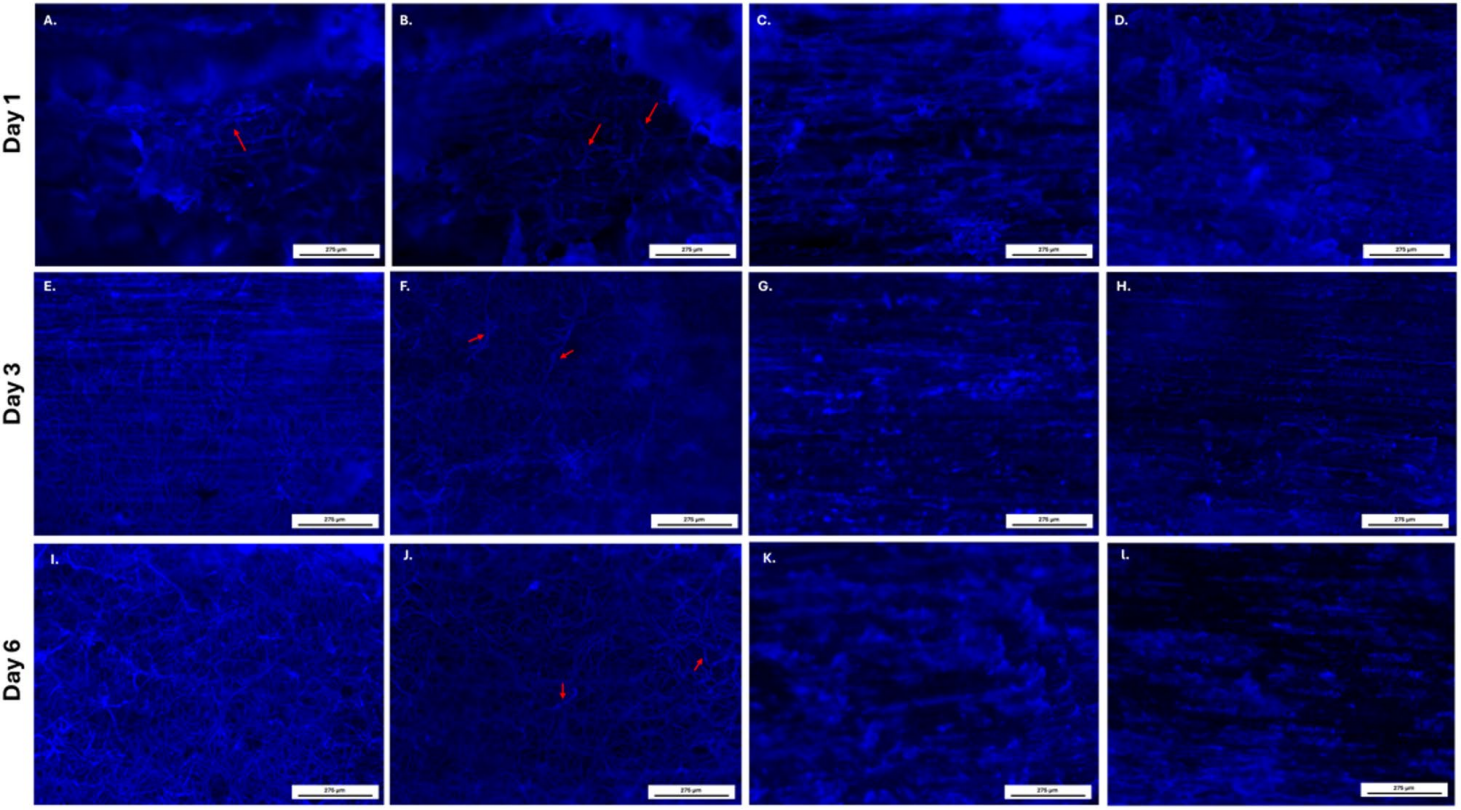
Fluorescent microscopy of wood pieces inoculated with or without *A. pullulans* EXF-3844 over days 1, 3, and 6. Day 1 (with *A. pullulans* **A**,**B**; controls** C**,**D**), day 3 (with *A. pullulans*: **E**,**F**; controls:** G**,**H**), day 6 (with *A. pullulans*: **I**,**J**; controls:** K**,**L**). Red arrows represent filamentous fungal structure. Images obtained under DAPI filter (Ex 350–400 nm, Em 425–475 nm) after CFW (0.3 mg/mL) staining.

VHX digital microscopy and CFW-based fluorescence imaging together revealed a progressive, time-dependent modification of the wood surface following *A. pullulans* inoculation (Figs. 4 and 5). At day 1, optical microscopy of wood pieces inoculated with *A. pullulans* revealed early signs of surface colonization (Fig. 4, sample A). The wood surface exhibited localized, faintly bright filamentous structures aligned across or perpendicular to the wood fibers (red arrows), indicating initial surface colonization of fungal elements. These features appeared discontinuous and sparsely distributed, with several thin, thread-like structures becoming distinguishable against the wood background. Fluorescence examination of CFW-stained samples further confirmed the presence of early surface-associated fungal structures (Fig. 5, samples A and B). Sparse, thin fluorescent elements were detected in localized regions (red arrows), consistent with limited initial colonization. By day 3, optical microscopical observations revealed a moderate increase in surface-associated structures compared with day 1 (Fig. 4, sample C). Thin, bright filamentous elements were observed in several regions along the wood pieces (red arrows), forming discontinuous but more extended patterns than those detected earlier. These structures remained relatively sparse and did not form continuous coverage across the field of view. Corresponding fluorescence images showed a rise in surface-associated fungal signal (Fig. 5, samples E and F). The fluorescent structures appeared brighter and more distinct than on day 1 (sample E), with localized filamentous networks visible aligned across or perpendicular to the wood fibers (red arrows in sample F). At day 6, optical microscopy revealed more prominent surface-associated structures on the inoculated wood compared with earlier time points (Fig. 4, sample E). Thin, bright filamentous elements were clearly visible along the wood fibers (red arrows), appearing more continuous than those observed on days 1 and 3. These structures remained unevenly distributed across the surface, forming localized regions of increased filament density. Fluorescence examination of CFW-stained samples demonstrated a stronger and more extensive fluorescent signal on the inoculated wood (Fig. 5, samples I and J). Filamentous structures were brightly delineated, forming dense, interconnected networks within several areas of the field of view (red arrows). Fluorescence intensity and spatial continuity were higher than those recorded on day 3, indicating a larger amount of surface-associated fungal material.

Across all observation time points, the control wood pieces consistently exhibited uniform surface textures in optical microscopy, with no detectable fungal filamentous material (Fig. 4, samples B, D and F). The appearance of the control surfaces remained unchanged throughout the incubation period, showing only the natural grain patterns characteristic of untreated pine wood. Similarly, with CFW–stained controls revealed no visible fungal fluorescent filaments at any time point, with all images displaying only background wood fluorescence (Fig. 5, Samples C, D, G, H, K and L). The absence of detectable features in both imaging modalities confirms that all observed surface-associated material in the inoculated samples resulted solely from *A. pullulans* exposure.

## Discussion

This study presents an integrated, multi-scale analysis of *A. pullulans* surface colonization on plastic and pine wood, enabling a detailed interpretation of how spatial expansion, and surface-associated biomass develop over time. By combining optical microscopy, including fluorescence observation, with quantitative fluorescence measurements, we obtained initial insights into the temporal sequence of attachment, spatial expansion, and surface coverage. The findings demonstrate the strong ability of *A. pullulans* to adhere to different substrates and highlight previously unreported features of its early structural organization, including the heterogeneous distribution of surface-associated filaments.

The coexistence of yeast-like blastoconidia and filamentous hyphal elements observed after 18 h of incubation fit with the well-known polymorphic growth behavior of *A. pullulans*. Previous studies have described *A. pullulans* as a dimorphic to polymorphic black yeast capable of simultaneously producing yeast-like cells and septate hyphae, with blastoconidia frequently arising from hyphal structures during early growth phases^5^.

It should be noted that cells used in the two experimental systems, plastic coverslips and wood were pre-cultured in different media, which may have influenced their physiological state prior to inoculation. For coverslip assays, cells were grown in SNB, a minimal medium that provides controlled conditions suitable for quantitative fluorescence measurements. In contrast, cells used for wood experiments were pre-cultured in PDB, a nutrient-rich medium that promotes higher biomass production before inoculation onto a heterogeneous substrate. While these choices reflect the specific requirements of each assay, they limit direct comparison between the two substrates and support interpreting the systems as complementary rather than directly equivalent models. Consistent with these substrate-specific experimental designs, the number of replicates differed between substrates due to the distinct nature of the experimental systems. For plastic coverslips, twelve replicates were used to enable robust quantitative fluorescence measurements under homogeneous conditions. In contrast, experiments on pine wood were performed with three independent replicates, as the analysis was primarily microscopy-based and aimed at capturing reproducible spatial colonization patterns on a structurally heterogeneous and anisotropic substrate.

Across plastic and wood surfaces, we found that *A. pullulans* initiate colonization through sparse, discontinuous filamentous structures at day 1, although notable variability in surface coverage was observed between replicates at this early stage, likely reflecting stochastic initial cell deposition and colonization events (Fig. 2—day 1). These structures subsequently develop into dense, interconnected layers by day 6. The increased vertical structuring and locally porous architecture observed at later stages on pine wood pieces (Figs. 4 and 5) may also be influenced by nutrient-driven hyphal extension^32^, although this mechanism was not directly assessed in the present study. However, because the two systems differ in inoculum volume, medium composition, and incubation configuration, the observed differences between substrates should be interpreted cautiously and not as a direct material-to-material comparison. This microscale sequence has not been previously described. Earlier studies reported the formation of functional biofilms on wood surfaces that contribute to protection and long-term surface stability, although characterization was mainly macroscopic and lacked insight into microscale surface colonization and structural development^12,28^. Moreover, results showed that the fungal colonization differed between the coverslip and wood assays, as evidenced by the formation of continuous surface-associated layers, and more uniform biomass distribution on plastic comparing to wood. This difference can be attributed to several interfacial properties intrinsic to the two materials. Plastic represent a smooth, non-porous, and chemically homogeneous polyester surface, providing a homogeneous and constant access to nutrients and hydration across the entire surface^33^, which minimizes physical barriers to attachment and enables expansion of adhering cells. In contrast, pine wood is a porous lignocellulosic substrate, where nutrient availability, moisture distribution, and surface accessibility are intrinsically heterogeneous^34^. These observations are consistent with interfacial studies showing that surface topography and wetting behavior strongly influence microbial attachment, with smoother and more uniform surfaces promoting faster biofilm establishment, whereas complex or heterogeneous microstructures tend to hinder adhesion^35^. The heterogeneous distribution of surface-associated filaments observed during early colonization may reflect a functional strategy that balances localized surface anchoring with exploratory growth^36^. Filamentous elements could serve as structural scaffolds that stabilize attachment, while yeast-like blastoconidia may facilitate rapid occupation of newly accessible surface areas. Such spatial heterogeneity may provide *A. pullulans* with a competitive advantage on heterogeneous substrates by allowing simultaneous persistence and expansion. Furthermore, quantification of *A. pullulans* colonization on plastic showed a marked decrease in fluorescence intensity at day 6, which likely reflects reduced dye penetration into compact, mature biofilms rather than an actual decline in biomass. The progression from sparse, discontinuous filaments at day 1 to dense, interconnected surface layers by day 6 suggests a temporally regulated colonization process rather than passive biomass accumulation. This transition is indicative of an early biofilm-like developmental program, characterized by increased spatial connectivity and surface coverage, which may enhance mechanical stability and resistance to environmental stress^37^. This interpretation aligns with previous studies reporting that the extracellular polymeric substance matrix of mature microbial biofilms can limit or completely block the diffusion of fluorescent dyes, leading to artificially reduced signals despite continued biomass accumulation^38^. Additionally, studies on bacterial biofilms have similarly demonstrated that as biofilms mature, their physicochemical properties, particularly wettability, surface roughness, and EPS accumulation, alter liquid interactions and hinder dye infiltration^39^. For example, wetting analyses of *Pseudomonas fluorescens* biofilms showed that high-nutrient, high-shear growth conditions produce surfaces that rapidly absorb droplets and exhibit complex dewetting behavior, reflecting increased thickness and structural heterogeneity^39^. These findings highlight methodological limitations when quantifying mature fungal biofilms using fluorescence measurements and underscore the need for complementary biochemical or imaging approaches for advanced time points.

The ability of *A. pullulans* to efficiently colonize both synthetic and natural substrates emphasizes its ecological versatility and explains its frequent presence on diverse built and natural environments^29^. On plastic surfaces, uniform colonization may facilitate persistent biofilm formation in anthropogenic settings, whereas the irregular growth observed on wood may reflect adaptive responses to microscale heterogeneity typical of natural lignocellulosic materials. The multimodal approach used in this study, combining optical microscopy, fluorescence imaging, and fluorescence-based quantification, was necessary because no single technique can fully capture the complexity of fungal colonization and early biofilm development. It should be noted that the present study does not provide direct measurements of adhesion strength, detachment resistance, or interfacial binding. Instead, the results reflect surface colonization dynamics inferred from microscopy and fluorescence-based biomass estimation. Similar conclusions were reached in recent interfacial studies of microalgal adhesion, where variations in surface chemistry and cell-substrate interactions produced distinct viscoelastic and structural responses that required complementary analytical tools for accurate interpretation^40^. In that work, quartz crystal microbalance with dissipation monitoring (QCM-D) enabled real-time detection of mass deposition and viscoelastic changes, revealing that surface free energy, zeta potential, and roughness strongly influenced biofilm formation^40^. These findings emphasize that quantitative fluorescence alone may not resolve the mechanical or structural properties of adhering biomass, particularly when EPS accumulation limits dye penetration, as observed on day 6 in our plastic assays. This limitation becomes even more pronounced on complex substrates such as wood, where quantitative fluorescence analysis was not feasible due to intrinsic heterogeneity, porosity, and autofluorescence, all of which compromise signal reliability. Accordingly, colonization on wood was assessed qualitatively, based on consistent spatial patterns observed across time points. Future analyses of *A. pullulans* adhesion would benefit from integrating label-free techniques such as QCM-D, optical profilometry, or atomic force microscopy, which can capture subtle mechanical transitions and provide continuous monitoring of attachment dynamics. Moreover, studies combining quantitative surface chemistry analyses (e.g., wettability, surface functional groups, and porosity) with molecular or biochemical markers of fungal adhesion and biofilm development could clarify the mechanistic basis of the substrate-dependent colonization patterns observed in *A. pullulans*^41^. Such complementary methodologies would strengthen the interpretation of surface–fungus interactions and support the development of predictive models for living-coating performance. However, the long-term stability and durability of *A. pullulans*-based surface colonization under environmental conditions were not assessed in the present study and represent an important direction for future work. Previous studies by our group have addressed these aspects and demonstrated the persistence and functional stability of similar living coatings under defined conditions. Evaluating coating persistence under controlled parameters such as humidity, temperature fluctuations, UV exposure, and nutrient limitation will be essential for translating these findings into practical applications.

In addition, while fluorescence-based quantification was successful on coverslips, direct measurement on wood remains challenging due to porosity, uneven absorption, and autofluorescence. Developing extraction-based methods, such as, enzymatic detachment^42^, sonication-assisted recovery^43^, or solvent-mediated extraction^44^ would enable accurate biomass quantification directly from wood. Such approaches will be essential for comparing biofilm formation across wood species, treatments, and fungal strains.

In the context of coatings on inert surfaces, interfacial properties such as surface energy, wettability, and chemical functionality are known to play a critical role in microbial attachment and biofilm development. Recent advances in surface-engineered systems have demonstrated that precise control of interfacial architecture, including amphiphilic structuring and polyelectrolyte-modified layers, can strongly influence interfacial interactions and functional performance^45–47^. These studies highlight the importance of tuning surface chemistry and structure to regulate biological or physicochemical processes at material interfaces.

Within this framework, the surface colonization of *A. pullulans* observed in the present study can be interpreted as the result of combined physicochemical and biological interactions. The production of extracellular polymeric substances, including pullulan and related polysaccharides, likely contributes to surface anchoring and structural cohesion^48^, while cell wall-associated components such as hydrophobins and melanin may modulate wettability, surface affinity, and resistance to environmental stress^49^. These features may also support surface persistence and potential resistance to fouling through matrix formation and space occupation, although these properties were not directly assessed in this study.

The methodological framework established in this study should be extended to evaluate *A. pullulans* colonization and adhesion on a wider range of building materials such as concrete, gypsum plaster, and metals (e.g., steel, aluminum). Such evaluation enables systematic comparisons of fungal colonization capacity across chemically and structurally distinct interfaces. Concrete is highly alkaline and porous^50^, which may limit early colonization due to pH stress while promoting capillary-driven moisture accumulation that could later facilitate filament penetration. Gypsum plaster is soft and hygroscopic^51^, potentially allowing deeper hyphal ingress but complicating imaging and detachment-based quantification. Metals vary widely in surface energy and corrosion behavior^52^, passivated surfaces (e.g., aluminum)^53^ may inhibit adhesion, whereas roughened or oxidized steel^54^ may provide micro-niches that enhance attachment. Comparative analysis across materials could reveal whether the early filamentous structures observed here represent a universal attachment strategy or are selectively induced by specific surface chemistry. Additionally, integrating these assays with targeted surface modifications, such as nutrient-limited conditions, would further clarify how *A. pullulans* sense and respond to interfacial cues. Such comparative studies are essential for predicting material susceptibility to colonization and for guiding the rational design of stable, biologically active coatings in engineered living materials applications.

## Conclusion

This study presents a multimodal characterization of *A. pullulans* surface colonization on plastic and pine wood, focusing on early-stage spatial organization and temporal development. Optical microscopy, fluorescence imaging, and fluorescence quantification revealed consistent temporal progression from sparse, discontinuous filamentous structures at early time points to more continuous and interconnected surface-associated networks over time.

On plastic surfaces, fluorescence-based measurements indicated an increase in signal from Day 1 to Day 3, followed by a decrease at Day 6, likely reflecting limitations in dye penetration within denser structures rather than a reduction in biomass. On wood, colonization exhibited greater spatial heterogeneity and appeared influenced by substrate microstructure, including fiber orientation and porosity. Due to the intrinsic differences between the experimental systems and the limitations of fluorescence-based quantification on porous substrates, the results should be interpreted as qualitative and semi-quantitative insights into surface-associated growth rather than direct measurements of adhesion strength or surface coverage.

Overall, this study highlights the importance of substrate properties in shaping fungal surface colonization patterns and demonstrates the value of combining complementary imaging approaches to investigate early biofilm development. These findings provide a basis for future studies aimed at quantitatively assessing fungal–surface interactions and evaluating their relevance in applied contexts such as living materials.

## Data Availability

Link of data on Zenodo: [https://doi.org/10.5281/zenodo.18300824](https:/doi.org/10.5281/zenodo.18300824).
